# Applying machine learning and natural language processing to patient safety event reports: Identifying patterns of cardiovascular diagnostic errors

**DOI:** 10.1371/journal.pone.0345693

**Published:** 2026-04-10

**Authors:** Azade Tabaie, Alberta K. Tran, Codrin Parau, Sonita S. Bennett, Sadaf Kazi, Kelly Smith, John Yosaitis, Kristen E. Miller

**Affiliations:** 1 Center for Biostatistics, Informatics, and Data Science, MedStar Health Research Institute, Washington, District of Columbia, United States of America; 2 Department of Emergency Medicine, Georgetown University Medical Center, Washington, District of Columbia, United States of America; 3 MedStar Health Research Institute, MedStar Health, Washington, District of Columbia, United States of America; 4 MedStar Health Institute of Quality and Safety, MedStar Health, Columbia, Maryland, United States of America; 5 National Center for Human Factors in Healthcare, MedStar Health Research Institute, Washington, District of Columbia, United States of America; 6 Institute of Health Policy, Management & Evaluation, University of Toronto, Toronto, Ontario, Canada; 7 Patient-Oriented Research, Michael Garron Hospital, Toronto, Ontario, Canada; 8 School of Medicine, Georgetown University, Washington, District of Columbia, United States of America; 9 MedStar Simulation Training & Education Lab, MedStar Institute for Innovation, Washington, District of Columbia, United States of America; National Research Council (CNR), ITALY

## Abstract

**Objective:**

To explore the utility of natural language processing (NLP) and machine learning (ML) techniques to identify unsafe conditions leading to cardiovascular diagnostic errors using patient safety event (PSE) reports data.

**Methods:**

PSE reports from January 2016 to August 2021 from a multi-hospital healthcare system in the mid-Atlantic region of the United States were included in this study. To have the true cardiovascular diagnostic errors labels for PSE reports, each individual PSE report was manually reviewed to find clinician-reported narratives describing current definitions of cardiovascular diagnostic errors. The PSE reports which contained cardiovascular diagnostic errors-related narratives were labeled as one and zero otherwise. Four binary ML models were employed to identify cardiovascular diagnostic errors narratives and common features from annotated PSE reports data: (1) simple logistic regression, (2) elastic net, (3) XGBoost, and (4) deep neural networks.

**Results:**

XGBoost outperformed the rest of the models in identifying cardiovascular diagnostic errors -related reports and achieved high performance metrics on the testing data (AUROC = 0.914, specificity = 0.982, PPV = 0.866, accuracy = 0.929, F-1 score = 0.738, and AUPRC = 0.783). Pacemaker emerged as a significant signal for cardiovascular diagnostic errors in our PSE reports. Our analysis demonstrated that ordering MRI for patients with pacemaker was a frequent theme among cardiovascular diagnostic errors-related PSE reports containing the word pacemaker. Order, EKG, cardiac, and chest were the five most important features in identifying cardiovascular diagnostic errors events. These words were utilized in explaining heart conditions, associated care process, and related safety incidents.

**Conclusions:**

Findings from our study demonstrates the feasibility of ML and NLP techniques in identifying cardiovascular diagnostic errors-related reports among the existing PSE data sources. However, validation in external healthcare systems is needed before broader application.

## 1 Introduction

Cardiovascular disease is the leading cause of morbidity and mortality in the United States (U.S.) [1]. Symptoms of cardiovascular disease can be subtle and manifest in a variety of forms, particularly in women and older adult populations, thus increasing their risk of cardiovascular diagnostic error [2–5]. The identification of diagnostic errors, including cardiovascular diagnostic errors, is an important part of safety surveillance and quality improvement. The National Academies of Sciences, Engineering, and Medicine (NASEM) recommended that healthcare organizations “monitor the diagnostic process and identify, learn from, and reduce diagnostic errors and near misses in a timely fashion” [6]. However, many safety programs that aim to detect diagnostic errors currently rely on manual reviews of free-text, narrative sources – such as patient safety event (PSE) reports or case reviews – which can be labor intensive and time consuming.

Several studies have found machine learning (ML) techniques to be effective in predicting diagnosis errors, including delayed and missed diagnosis. For example, Bhasuran et al. utilized ML models with electronic health records (EHR) data to identify undiagnosed patients with a rare but treatable disease [7]. In another study, ML was used to classify cases of accurate delirium diagnosis vs. misdiagnosis among patients who were diagnosed by consultation-liaison psychiatry [8]. Moreover, ML models have a broad range of applications in identifying markers for delayed cancer diagnosis [9–11]. However, and despite the application of ML models to detect cardiovascular disease [12,13], to our knowledge, ML techniques have not yet been evaluated in identifying patients at higher risk of cardiovascular diagnostic errors. While identifying risk of cardiovascular diseases is a crucial part of patients’ care plans and clinical trajectories, a further understanding of contributors to cardiovascular diagnostic errors can inform efforts and interventions to avoid the serious patient health consequences that can result from these missed or delayed diagnoses [14].

Many health organizations have safety systems that utilize PSE reports, allowing for healthcare providers to anonymously submit free-text descriptions about near misses or safety problems within clinical settings to enhance safety surveillance and learning. Natural language processing (NLP) and ML approaches have been used with increasing success to analyze the rich information in PSEs into digestible categories to motivate solutions. In a recent study, NLP and ML techniques were applied to a PSE reporting system to sort recorded medication errors into more commonly utilized categories (e.g., wrong drug, wrong time, wrong strength or concentration, etc.) [15]. In another study, Chen et al. investigated the efficacy of ML models in automatically classifying different event types such as care coordination or communication, laboratory test, medication related, etc. using PSE reports from maternal encounters and mother-baby units [16]. Fong et al. applied ML techniques to free-text formatted data within a PSE reporting health system comprised of ten hospitals to identify safety incidents with narratives related to health information technology [17,18]. To our knowledge, there are no published studies that incorporates ML techniques and PSE reports to identify cardiovascular diagnostic errors. However, health system efforts to improve diagnostic capacity and other quality and safety issues typically include manual case-by-case reviews and analysis of PSEs and other similar safety reports. For example, Measure Dx, a publicly available resource developed by the Agency for Healthcare Research and Quality to provide guidance for healthcare organizations to identify, analyze, and learn from diagnostic safety events, found that most organizations in their project re-examined existing quality and safety data that had captured previously identified safety events; however, more than half of the organizations in their sample utilized an EHR-enhanced chart review strategy to identify high-risk diagnoses or care patterns suggestive of missed opportunities and/or to bolster manual chart review strategies [19]. Therefore, a further investigation of advanced methodologies to help sort through PSE reports and other similar existing data can help pave the way for healthcare organizations to more efficiently identify and respond to diagnostic errors of cardiovascular disease and other diseases, and improve care delivery and quality.

While PSE reports are rich, commonly utilized data sources within most healthcare systems, the lack of actual consistent diagnostic error labels limit the application of ML and predictive modeling in identifying patients who experienced diagnostic errors broadly, and particularly for specific diseases. For instance, the following PSE report narrative was identified as an explanation of a delayed cardiovascular diagnosis event. However, making the connection to cardiovascular diagnostic errors requires manual reading of each PSE report:

“*Patients are always sent back without the telemetry monitor from MRI, and if the patient had an arrhythmia, it would not be noticed due to being off the monitor for an extended period due to delays with transporting patient back and forth*”.

In this study, with a team of clinicians, researchers, and diagnostic safety experts, we annotated a large body of PSE reports and identified reports with cardiovascular diagnostic errors narratives. Then using the annotated dataset with true labels (i.e., cardiovascular diagnostic errors vs. non-cardiovascular diagnostic errors), we trained binary ML models to classify PSE reports that contained similar narratives. Statistically significant differences among demographic features of patients with and without cardiovascular diagnostic errors were investigated. Significant terms within PSE reports that were associated with cardiovascular diagnostic errors were detected. While we used well-established NLP and ML approaches, our contribution is the novel application of these techniques to detect disease-specific diagnostic errors within PSE reporting system data, a data source not traditionally leveraged for diagnostic safety surveillance. This translational approach aims to bridge methodological advances in data science with operational needs in patient safety and quality improvement.

## 2 Materials and methods

### 2.1 Data source

The data source was PSE reports categorized under the general event type of *diagnosis/treatment* or *diagnostic imaging* from January 2016 to August 2021 from a multi-hospital healthcare system in the mid-Atlantic region of the U.S. General event type is a part of PSE report that identifies the overall theme for the near misses or safety problems in the clinical setting (e.g., fall, blood product, healthcare IT, etc.). We focused on PSE reports with general event type of *diagnosis/treatment* or *diagnostic imaging* that may contain information regarding cardiovascular diagnostic errors. Reports with empty or insufficient brief factual description (e.g., “100cc omni 350 infiltrated in right bicep area”) were removed prior to manual reviews. Medical record number (MRN), date of birth (DOB), severity level, event date, brief factual descriptions, and reported contributing factors were also extracted from each PSE report for further descriptive analysis. Patients’ demographics information is not commonly recorded in PSE reports. Therefore, PSE reports were cross matched with EHR data, using MRN and DOB, to capture patient demographics such as sex, race, ethnicity, and primary language.

### 2.2 Cardiovascular diagnostic errors labels

Using the definition of diagnostic errors from the NASEM’s *Improving Diagnosis in Healthcare* report [20], we defined cardiovascular diagnostic errors as any delay in diagnosis/treatment or misdiagnosis of a cardiovascular-related condition. To confirm ground truth (i.e., true cardiovascular diagnostic error labels) for the supervised ML algorithm to learn patterns in the data, we annotated free-text descriptions from PSE reports and manually reviewed 7,467 reports to identify “truth” indications of cardiovascular diagnostic errors.

Data annotation of PSE reports/free-text datasets typically involves manual review of the free-text data to investigate the presence of specific narratives [21]. For instance, in this study, data annotation was conducted to detect narratives of diagnostic errors in the free-text part of PSE reports. To obtain the true cardiovascular diagnostic errors labels for PSE reports, a research associate specialized in human factors engineering and familiar with PSE reports manually reviewed the 7,467 PSE reports included in this study using key definitions from the NASEM’s report [20]. For this study, a cardiovascular event was defined as any incident that could potentially cause damage to the heart muscle or surrounding vascular structures, a definition derived from general medical literature on cardiac and cardiovascular events. With the guidance of a clinical expert team, the research associate reviewed all PSEs that were categorized as diagnostic-related events for whether they 1) met the NASEM definition of diagnostic error and 2) included a keyword or process related to cardiovascular disease. The identified keywords and phrases within PSE reports that could be considered a cardiovascular diagnostic error included but not limited to chest, DVT, echo, BP and pacemaker. Any reports that required more in-depth clinician review or clinical understanding were labeled as “unclear” and were reviewed by two clinical subject matter experts. For instance, vascular issues like deep vein thrombosis and pulmonary embolism were not initially categorized as cardiovascular events, but after consultation with the clinical team, were intentionally included in our review to align with clinicians’ clinical framing and conceptualizations around potential cardiovascular consequences. Table 1 summarizes the diagnosis error categories that were included in the manual review of the PSE reports. Additional categories, such as ‘good catch’ (i.e., an event that could have led to significant harm but was prevented in time) and ‘delay of treatment’ (i.e., events related to treatment rather than diagnosis errors), were included in training the ML models but were not considered diagnostic errors in this study.

**Table 1:** Categories of cardiovascular diagnostic errors. Different cardiovascular diagnostic errors were incorporated in identifying PSE reports with such narratives. De-identified examples of PSE reports were included.

Categories of cardiovascular diagnostic errors	Definition	Example
Missed diagnosis	When a diagnosis is overlooked or not considered.	Patient was admitted from ER for lower GI bleed, anemia and blood in stool. patient was to be placed on non-cardiac tele, but no indication was provided. patient was assigned a med-surg bed and was brought up to the floor. Soon after arrival, orders to transfer patient to IMC was placed. This was due to patient’s extensive cardiac history and patient was tachycardia in the ED.
Delayed diagnosis	When a diagnosis is identified but not promptly communicated or acted upon.	When consult was seen and it was confirmed that the patient only needed IV access, no true need for a PICC line. It was stated that the patient lost IV access the day prior, and the decision was made to wait for IR to place a PICC line. it is concerning that this patient, who required ICU monitoring and care, did not have IV access for a prolonged period of time. This was an inappropriate PICC line consult.
Wrong diagnosis	When an incorrect diagnosis is made.	No example in our data.
Failure to recognize complications	When a correct diagnosis is made, but complications are not recognized, worsening the patient#39;s condition.	Doctor A was getting ready to flip the patient with the surgical residents. the blood pressure was 90s/60s, then 61/27. The blood pressure was 120/74 at flip. Doctor A disconnected from the anesthesia machine and switched to the portable monitor. Doctor B came in saw the patient#39;s heart rate was in the 80s which was bradycardic considering the patient#39;s heart rate was in the 140s and then finally down to 100 and teens. He could not feel a pulse but saw the electrical activity and asked that we start chest compressions and 1 mg of epinephrine was given. A total of 3 cycles of chest compressions and 1 mg of epinephrine was given, a pulse was regained, patient was stabilized.
Failure to diagnose an unrelated disease	When an unrelated condition is missed because the clinician focuses solely on the primary diagnosis.	Patient returning from CVICU earlier in day. Patient was to transfer according to EHR order. RN saw glucose was low and was not mentioned during transfer report. RN called CVICU RN to question. CVICU RN stated apple juice was given to patient. This event occurred close to change of shift timing.

**Fig 1:**
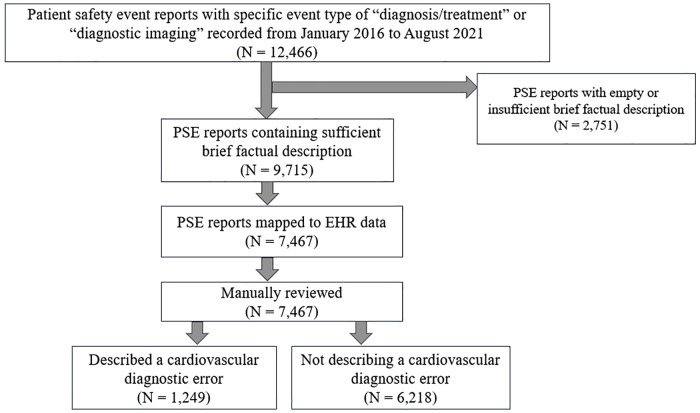
A graphic representation of the quantitative data analysis approach for capturing cardiovascular diagnostic errors from patient safety events.

PSE reports that contained a narrative indicating a cardiovascular diagnostic error from definitions in Table 1 were labeled as one (or labeled zero otherwise). Fig 1 summarizes our methods to identify and label PSE reports indicating a cardiovascular diagnostic error.

To assess interrater reliability (IRR) of the manual annotation process, a random 1% sample (n = 75) of the reviewed reports was independently annotated by a second reviewer with experience in clinical data annotation, who was blinded to the original labels assigned by the first reviewer. The IRR between the two reviewers was 90%. Among the sampled reports, 10 were labeled as describing cardiovascular diagnostic errors by the first reviewer, and all 10 were identically labeled by the second reviewer. Two additional reports, originally labeled as non-cardiovascular diagnostic errors, were identified by the second reviewer as describing cardiovascular diagnostic errors; one report described communication gaps between cardiac surgical teams that delayed surgery, and another described a mismatch in BiPAP machine sizing. These findings suggest a high level of consistency between reviewers and support the validity of the annotation process.

### 2.3 Statistical tests

Wilcoxon rank-sum test for numerical features and Chi-squared test for categorical features were applied to evaluate any statistically significant differences of patient characteristics (e.g., age, sex, race) between patient groups with and without cardiovascular diagnostic errors.

### 2.4 Machine Learning (ML) models

#### 2.4.1 Feature extraction from free-text data.

The unstructured free-text descriptions of PSE reports that were submitted by clinicians served as the input of the classification models. The free-text data was transformed to lower case. Punctuations, extra white spaces, stop words, and numbers were removed, and the words were tokenized. We calculated Term Frequency-Inverse Document Frequency (TF-IDF) features from these transformed free-text data. TF-IDF feature extraction was selected due to its simplicity, computation efficiency, and ease of interpretation [22]. More advanced NLP approaches, such as contextual embeddings (e.g., BERT, ClinicalBERT), were not applied in this study because they require substantially greater computational resources and introduce additional complexity. Our aim was to determine whether meaningful signal could be extracted from PSE reports using TF-IDF before scaling to transformer-based models in future work.

#### 2.4.2 Training the ML models.

PSE reports were analyzed at the report level rather than the patient level because a patient may have multiple PSE submissions and patient identifiers were not consistently available across all reports. Each PSE record has a unique report ID, and aggregation across patients was not feasible.

Train and test data splits were done through a random stratified sampling. Eighty percent of the data was used as training set and the rest were utilized to test the classifiers. Given the moderate class imbalance in our dataset (approximately 17% of PSE reports containing cardiovascular diagnostic errors), we evaluated multiple strategies to mitigate bias, including oversampling of the minority class, undersampling of the majority class, and application of class weights. While oversampling and undersampling improved training sensitivity, these methods reduced the generalizability of the models on the testing data. Therefore, we opted to set the model sensitivity threshold to 0.8 to ensure comparability across models and maintain external validity. We tested four types of ML classification models to classify PSE reports indicating cardiovascular diagnostic errors: (1) simple logistic regression, (2) logistic regression model with L1L2 or elastic net regularization, (3) eXtreme Gradient Boosting (XGBoost), and (4) feed-forward deep neural networks with binary cross-entropy loss function (DNN) [23–26]. Simple logistic regression model was employed as the baseline model for comparison in lieu of a similar ML work in the literature [22].

The hyperparameters of the classification models were optimized using different optimization methods (Bayesian optimization, cross-validation). Appendix A contains the list of hyperparameters, the searched ranges, and stopping criteria. To evaluate the performance of the trained models, we calculated area under operative characteristic curve (AUROC), sensitivity, specificity, positive predictive value (PPV), negative predictive value (NPV), accuracy, F-1 score, and area under the precision–recall curve (AUPRC).

#### 2.4.3 Feature importance.

SHapley Additive exPlanations (SHAP) algorithm which is a method for explaining predictive models based on game theory was used to capture features that influenced the decision making of the best-performing model [27]. SHAP values presents the contribution of each feature to the model’s decision-making process and their effect size on the predicted outcome (e.g., cardiovascular diagnostic errors). The features with a higher value in model’s decision-making process have higher mean absolute SHAP values.

### 2.5 Ethical consideration

This study was approved by our Institutional Review Board (IRB #00001245) on 26/08/2019 and the approved protocol has been active since then. This study was conducted between 26/08/2019 and 31/12/2024. During this period, our team accessed and analyzed PSE reports. No patient or provider identifiers were queried or used in this research.

## 3 Results

### 3.1 Descriptive analysis

A total of 12,466 PSE reports were queried from the database. Of these, 2,751 (out of 12,466, 22.1%) PSE reports had empty or insufficient brief factual descriptions, and were removed prior to data annotation. A total of 9,715 (out of 12,466, 77.9%) PSE reports had brief factual descriptions recorded. Of these, 7,467 (out of 9,715, 76.9%) PSE reports that were associated with 6,999 unique patients were successfully mapped to EHR data, then manually reviewed. Our analyses were conducted using the 7,467 PSE reports which could be mapped to EHR data. There were 1,249 PSE reports (out of 7,467, 16.7%) associated with 1,124 unique patients (out of 6,999, 16.1%) that contained cardiovascular diagnostic errors-related narratives. Table 2 represents the study cohort and PSE reports characteristics.

**Table 2:** Characteristics of the Study Sample. The denominator to calculate percentages are the number of unique patients in each group (i.e., all patients = 6,999; patients with cardiovascular diagnostic error = 1,124; and patients without cardiovascular diagnostic error = 5,875). The percentages for severity level do not sum up to 100% as one patient may have had multiple PSE reports each recorded with different categories of severity level. The single asterisk demonstrates statistically significant difference (i.e., p-value < 0.05) between patients with and without cardiovascular diagnostic error. Statistical comparisons are unadjusted and intended for exploratory interpretation only.

Patients Characteristics				
Characteristics	All Patients (n = 6,999)	With Identified Cardiovascular Diagnostic Error ^a^ (n = 1,124)	No Cardiovascular Diagnostic Error ^a^ (n = 5,875)	p-value
**Number of Unique Patients**	6,999	1,124 (16.06%)	5,875 (83.94%)	
**Age in Years** Mean (standard deviation)	58.26 (20.74)	62.34 (19.41)	57.44 (20.98)	< 0.001*
**Sex** N (%)				
Female	3,767 (53.82%)	590 (52.49%)	3,177 (54.08%)	0.33
Male	3,232 (46.18%)	534 (47.51%)	2,698 (45.92%)	
**Race** N (%)				
White	3,217 (45.96%)	484 (43.06%)	2,732 (46.5%)	0.008*
Black	3,182 (45.46%)	557 (49.56%)	2,624 (44.66%)	
Other	600 (8.58%)	83 (7.38%)	517 (8.84%)	
**Ethnicity** N (%)				
Hispanic or Latino	120 (1.71%)	12 (1.07%)	108 (1.84%)	0.19
Not Hispanic or Latino	6,530 (93.3%)	1,055 (93.86%)	5,475 (93.19%)	
Unknown	349 (4.99%)	57 (5.07%)	292 (4.97%)	
**Primary Language** N (%)				
English	6,783 (96.91%)	1,099 (97.78)	5,684 (96.75%)	0.08
Spanish	100 (1.43%)	10 (0.89%)	90 (1.53%)	
Sign Languages	9 (0.13%)	3 (0.27%)	6 (0.1%)	
Other	107 (1.53%)	12 (1.06%)	95 (1.62%)	
**PSE Reports Characteristics**				
**Characteristics**	**All PSE Reports**	**With Identified Cardiovascular Diagnostic Error** ^**a**^	**No Cardiovascular Diagnostic Error** ^**a**^	**p-value**
**Number of PSE Reports**	7,467	1,249 (16.73%)	6,218 (83.27%)	
**Severity Level** N (%)				
No harm	686 (9.8%)	96 (8.54%)	590 (10.04%)	**0.27**
Harm	6,307 (90.11%)	1,032 (91.81%)	5,272 (89.74%)	
Death	64 (0.91%)	10 (0.89%)	54 (0.92%)	

^a^Determined by reviewer.

### 3.2 Performance of ML models

**Fig 2:**
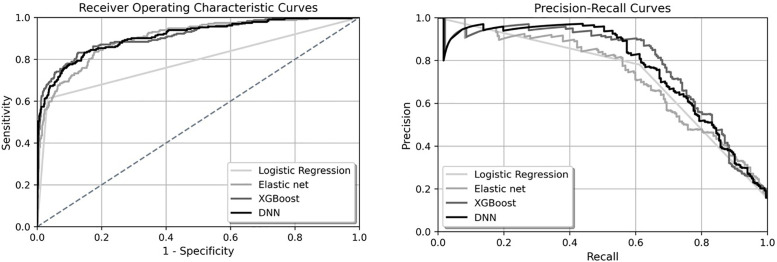
Receiver Operating Characteristics (ROC) Curve and Precision-Recall (PRC) Curve of Classifying PSE Reports with Cardiovascular Diagnostic Error Using TF-IDF Feature Matrix.

Appendix B contains the list of TF-IDF features. The performance of four ML models is presented in Table 3. XGBoost outperformed the rest of the models with AUROC of 0.914, specificity of 0.982, PPV of 0.866, accuracy of 0.929, F-1 score of 0.738, and AUPRC of 0.783 on the testing data. Elastic net was a more sensitive model and achieved the sensitivity of 0.711 among all trained models. The simple logistic regression model achieved 100% sensitivity and 0% specificity on the test set, indicating that it classified all reports as positive for cardiovascular diagnostic error. This degenerate solution highlights the limitations of linear models without regularization when applied to imbalanced, high-dimensional text data. While included as a baseline, this model is not interpretable or clinically useful. Fig 2 demonstrates the superior performance of XGBoost model in terms of AUROC and AUPRC. Appendix C includes the optimized hyper-parameters values for the XGBoost model.

**Table 3:** Performance of Classifying PSE Reports with Cardiovascular Diagnostic Error Using TF-IDF Feature Matrix. Bold fonts demonstrate the best value achieved for each performance metrics. The numbers in brackets represent the estimated 95% confidence intervals.

Metrics	Simple Logistic Regression		Elastic Net		XGBoost		DNN	
**Train**	**Test**	**Train**	**Test**	**Train**	**Test**	**Train**	**Test**
**AUROC**	0.828 [0.812, 0.843]	0.79 [0.79, 0.79]	0.929 [0.921, 0.938]	0.902 [0.903, 0.903]	0.981 [0.978, 0.985]	**0.914** [0.914, 0.914]	0.958 [0.951, 0.965]	0.911 [0.911, 0.911]
**Sensitivity**	1 [1,1]	1 [1,1]	0.8 [0.8, 0.806]	0.711 [0.694, 0.733]	0.8 [0.8, 0.806]	0.642 [0.634, 0.66]	0.802 [0.8, 0.805]	0.677 [0.647, 0.703]
**Specificity**	0 [0, 0]	0 [0, 0]	0.907 [0.881, 0.926]	0.898 [0.872, 0.913]	0.989 [0.983, 0.991]	**0.982** [0.975, 0.983]	0.964 [0.954, 0.973]	0.95 [0.936, 0.964]
**PPV**	0.155 [0.155, 0.155]	0.155 [0.155, 0.155]	0.613 [0.552, 0.664]	0.561 [0.514, 0.594]	0.927 [0.897, 0.944]	**0.866** [0.828, 0.871]	0.802 [0.76, 0.847]	0.714 [0.668, 0.765]
**NPV**	0 [0, 0]	0 [0, 0]	0.961 [0.96, 0.962]	**0.944** [0.941, 0.947]	0.964 [0.964, 0.965]	0.937 [0.936, 0.94]	0.964 [0.963, 0.964]	0.941 [0.937, 0.945]
**Accuracy**	0.155 [0.155, 0.155]	0.155 [0.155, 0.155]	0.891 [0.868, 0.906]	0.869 [0.851, 0.879]	0.959 [0.955, 0.962]	**0.929** [0.926, 0.93]	0.939 [0.93, 0.947]	0.908 [0.9, 0.916]
**F-1 Score**	0.268 [0.268, 0.268]	0.269 [0.269, 0.269]	0.694 [0.653, 0.726]	0.627 [0.607, 0.64]	0.859 [0.846, 0.867]	**0.738** [0.733, 0.74]	0.802 [0.78, 0.824]	0.695 [0.685, 0.711]
**AUPRC**	0.797 [0.778, 0.817]	0.726 [0.726, 0.726]	0.762 [0.739, 0.788]	0.726 [0.726, 0.726]	0.926 [0.913, 0.94]	**0.783** [0.783, 0.783]	0.862 [0.843, 0.882]	0.774 [0.774, 0.774]

To evaluate a decrease in the number of features used in training the ML models, we also applied Chi-square feature selection with TF-IDF features and compared them with the other models. However, we found that approach did not improve the classification performance (Appendix D). Moreover, integrating patients’ demographic with TF-IDF features did not boost the classification performance of the XGBoost model (Appendix E).

### 3.3 Most important features

**Fig 3:**
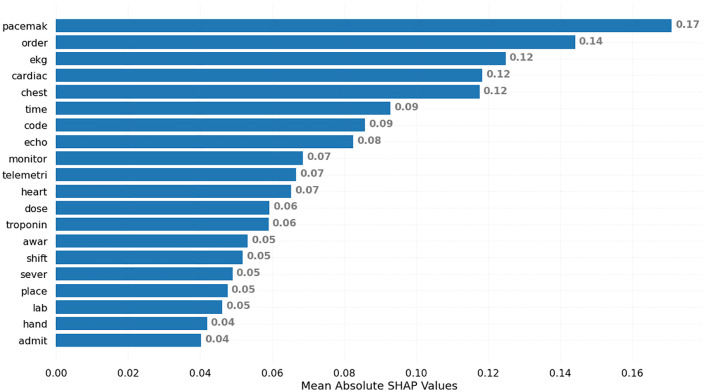
Mean SHAP values plot. For each feature, we calculated the mean SHAP value across all observations. Specifically, we take the mean of the absolute values as we do not want positive and negative values to offset each other. Features that have made large positive/negative contributions will have a large mean SHAP value. In other words, these are the features that have had a significant impact on the model’s predictions. Words were stemmed.

Fig 3 demonstrates the features that were most influential in identifying the reports with cardiovascular diagnostic errors within the sample of PSE reports. Pacemaker (SHAP = 0.17), order (SHAP = 0.14), EKG (SHAP = 0.12), cardiac (SHAP = 0.12), and chest (SHAP = 0.12) were the five most important features in the decision-making of the XGBoost model, respectively.

## 4. Discussion

Cardiovascular diagnostic errors were identified in 16.1% of reviewed PSE reports. To our knowledge, our study is the first to evaluate cardiovascular diagnostic error from PSE reports. In lieu of an estimated prevalence rate of cardiovascular diagnostic errors from PSE reports in the literature, we compared the cardiovascular diagnostic errors rate from our data to the CRICO Strategies Comparative Benchmarking System (CBS) which is the largest malpractice claims database with detailed coding in the world, containing  >  350,000 medical malpractice cases from outpatient general medicine. The prevalence of cardiovascular diagnostic errors was reported as 10% in CBS database [28], which is lower than what we found in PSE reports. However, our data included both outpatient and hospital settings, which may have led to the higher rate of cardiovascular diagnostic errors in our study.

We observed statistically significant associations between age, race, and the presence of cardiovascular diagnostic error–related narratives in our PSE report sample, with a higher proportion of identified diagnostic error narratives reported in older Black patients. Our observations are consistent with the literature, with a recent systematic review describing consistent increased risk for missed diagnosis of myocardial infarction and acute coronary syndrome among Black patients in emergency department settings [29]. One potential explanation for this may be due to longer wait times for physician evaluations observed in historically marginalized patient subgroups. For example, a recent study of emergency department visits for adults between 18–55 years of age in the National Hospital Ambulatory Medical Care Survey found that women and people of color waited longer for physician evaluations, independent of their clinical features [30]. Overall, these demographic differences should be interpreted as associative patterns rather than causal relationships and particularly with the use of clinician-reported data. Clinician-reported data are best understood as capturing signals of diagnostic error and are more suitable for surveillance, learning, and hypothesis generation than for estimating true diagnostic error rates. As such, our findings may reflect systemic, contextual, or reporting-related factors within the healthcare system rather than inherent disparities in diagnostic accuracy. Further, the observed differences in race distributions between cardiovascular diagnostic error and non-error groups in Table 2 highlight the importance of evaluating algorithmic fairness. Variations in how patient safety events are documented and reported across different demographic groups could influence both model features and performance. While our current evaluation focuses on overall accuracy, future work will incorporate subgroup fairness analyses, such as stratified sensitivity and specificity estimates and fair-ML metrics (e.g., equal opportunity difference), to ensure that performance is equitable across demographic groups and that automated identification methods do not inadvertently reinforce existing disparities in diagnostic safety.

Findings from our study demonstrate the potential for health systems to capture disease-specific diagnostic errors from existing PSE data sources, and further accelerate diagnostic safety learning and improvement efforts. Although our study focused on cardiovascular diseases only, future studies can use similar approaches to identify diagnostic errors related to other diseases and utilizing existing PSE data. We found that XGBoost model outperformed the other models in identifying patients at higher risk of cardiovascular diagnostic errors. Similar studies looking at ML models have also found that this algorithm works better than linear models such as logistic regression and commonly works better than DNN models in classifying tabular data, like TF-IDF features in this study [31,32]. Moreover, DNN models require large training dataset while XGBoost can conveniently be trained on small to medium sized data. XGBoost is highly flexible and customizable making it well-suited for classification tasks with high-dimensional feature space and class-imbalanced data.

The XGBoost model achieved a sensitivity of 64.2%, indicating that approximately one-third of cardiovascular diagnostic error–related reports were not identified. This miss rate may be too high for sole use in patient safety surveillance, where sensitivity is often prioritized to avoid overlooking potential diagnostic concerns. However, as a proof-of-concept triage tool, the model could still provide value by substantially reducing the volume of reports requiring manual review, with the understanding that it supplements rather than replaces human review. Acceptable sensitivity thresholds ultimately depend on the system’s intended use case. Adjusting probability thresholds could increase sensitivity, but doing so would come at the cost of specificity and increased reviewer workload. Further threshold optimization and clinical validation will be essential in future work.

We observed performance differences between the training and testing sets (e.g., XGBoost sensitivity decreasing from 0.80 to 0.642 and PPV from 0.927 to 0.866). These gaps likely reflect a degree of expected overfitting, given the high dimensionality of the TF-IDF feature space and the linguistic variability inherent in narrative safety reports. Although cross-validation, regularization, and Bayesian optimization were used to mitigate overfitting, these results underscore the importance of external validation and suggest that the model may be learning system-specific terminology. Future work will explore additional regularization strategies and evaluate performance on PSE reports data from other healthcare systems.

It is important to note that the methodological components of this study (TF-IDF feature extraction and standard supervised learning classifiers) were intentionally selected for their interpretability and reproducibility rather than algorithmic novelty. The innovation of this work lies in demonstrating that these established techniques can be potentially operationalized within healthcare safety infrastructures to automatically identify cardiovascular diagnostic error narratives, a previously untested application that supports scalable diagnostic safety surveillance.

According to the mean absolute SHAP values, *pacemaker* emerged as a significant signal for cardiovascular diagnostic errors in our PSE reports. Further analysis of the free-text descriptions of reports containing the word *pacemaker* suggested that a frequent theme involved issues around ordering or completing MRIs for patients with or incorrectly suspected of having pacemakers. For example, delays in patients completing MRI scans were reported in PSE reports, describing scenarios where key MRI screening information was inaccurate or not communicated, which delayed the test results and diagnosis process. For instance, “*Patient was ordered for an MRI two days ago. MRI still has not been completed. This morning when MRI was called, they had the wrong information that the patient has a pacemaker, which the patient does not have. This is a delay in patient care as this was ordered two days ago*”. Additional features with high mean absolute SHAP values included *order*, *EKG, cardiac, and chest*. Most of the words with high SHAP values can be used by health systems to warrant further exploration around patients’ heart conditions, associated care processes, and related safety incidents. It is important to emphasize that the relationships identified between textual features (e.g., pacemaker, order, EKG) and cardiovascular diagnostic errors reflect associative patterns captured by the model rather than causal mechanisms. The observed feature importance values indicate terms statistically associated with diagnostic error narratives within PSE reports but should not be interpreted as evidence of causal pathways or contributing factors.

Although our exploratory review found that many pacemaker-related PSE reports involved delays or confusion surrounding MRI ordering and device compatibility, this was intended as an illustrative example rather than a comprehensive sub-analysis. Pacemaker-related reports likely represent several distinct failure modes, including inaccurate or incomplete MRI screening information, documentation errors about device status, uncertainty regarding device compatibility, and breakdowns in communication across clinical teams. A systematic qualitative and quantitative analysis of these reports (e.g., quantifying the proportion attributable to MRI-related issues vs. other process failures) would provide deeper insights but was beyond the scope of this proof-of-concept study. Future work will develop a structured taxonomy to categorize these failure modes and evaluate targeted interventions, such as standardizing MRI-screening workflows, improving documentation of implanted devices, and enhancing communication pathways around cardiac device management.

In its current form, this model should be viewed as a proof-of-concept screening tool intended to support and not replace manual PSE review processes. In many healthcare systems, PSE reports are manually reviewed by clinicians, patient safety officers, quality staff, or human factors specialists, a process that can be time-consuming given the volume of reports and the relative rarity of diagnostic error–related events. An automated classifier could serve as an initial triage step by prioritizing reports with language patterns associated with cardiovascular diagnostic errors. Reports flagged by the model would then undergo traditional manual review by clinical and safety experts, ensuring that human judgment remains central. This triage approach has the potential to reduce reviewer workload, focus attention on potentially higher-risk narratives, and accelerate organizational learning cycles. However, any operational deployment would require external validation, assessment of workflow integration, and evaluation of potential time or resource savings.

Our study has some key limitations. First, although the PSE reports came from several different hospitals, all hospitals were part of the same healthcare system and therefore may reflect specific language, format, or shared cultural values that are unique to this healthcare system and thus may not be generalizable to other hospitals and healthcare organizations. The developed ML classifier should be validated on data from multiple hospitals to validate generalizability. Accordingly, this study should be interpreted as a proof-of-concept requiring external validation before clinical or operational use. Future work will focus on external validation using PSE reports from other healthcare systems to assess the reproducibility and robustness of the model across diverse reporting environments. Differences in event taxonomies, narrative styles, and safety reporting processes may affect model performance, and validating the approach in heterogeneous datasets will be critical to ensure generalizability and facilitate widespread implementation within diagnostic safety monitoring workflows. Second, since the number of PSE reports for review was high and the review process was labor intensive, we utilized a full-time, non-clinically based research associate to manually review each individual PSE report and assign cardiovascular diagnostic errors labels. While the research associate was specialized in human factor engineering and experienced with health systems safety analyses, reviewer bias could have been introduced throughout the review process. To reduce this bias, the reviewer was trained to identify “unclear” reports that were further reviewed in tandem with clinical experts. Also to further enhance the credibility of the labeling process, we performed an IRR assessment on a random subset of reports. The observed 90% Cohen’s Kappa between the two reviewers supports the reliability of the manual annotations used for model training and evaluation. However, future studies could consider the use of a larger sample of the reports to be reviewed by a second reviewer to reduce potential reviewer biases in the coding schema. Third, there are indeed a large number of classifiers that could be trained on this problem; however, to maintain clarity and focus, we intentionally selected a concise set of models that are most appropriate for text-based, imbalanced data and that represent different methodological families (linear, regularized, ensemble, and neural network). Future work should expand the ML classifiers applied on this problem to find other potentially well-suited models. Finally, this study utilized the TF-IDF approach for feature extraction from free-text data. This method was chosen as an effective initial step for exploring the informational content embedded in narrative reports. As part of text preprocessing, numbers and stop words were removed to reduce noise in the TF-IDF feature space. This step is appropriate for TF-IDF because such models rely on raw term frequencies and do not capture semantic or contextual relationships. We acknowledge that in more advanced NLP methods (e.g., BERT variants), numerical values, multi-word medical phrases, and contextual cues may carry clinically meaningful information and would therefore be retained in those models. Although intermediate approaches such as n-grams or contextual embeddings may improve model performance, our objective in this study was to demonstrate a proof-of-concept using interpretable, computationally efficient feature representations. Future work using transformer-based approaches will incorporate these elements. More advanced techniques, such as BERT-based models and large language models (LLMs), present a promising direction for future exploration to uncover deeper contextual patterns within PSE narratives. At present, evidence supporting their performance in PSE reports is limited, and further research is needed to evaluate their feasibility, accuracy, and interpretability in this domain before clinical or operational implementation. Additionally, future studies could expand the scope of analysis by incorporating data sources beyond PSE reports, including problem lists, ICD-10 codes, and discharge summaries. Integrating these data sources may enhance the identification of specific missed or delayed cardiovascular diagnoses.

Lastly, as different operational use cases require different performance trade-offs, future implementations should explore threshold tuning to align model outputs with the needs of patient safety teams. A comprehensive evaluation of sensitivity–specificity trade-offs will be required before integrating this model into practice.

## 5 Conclusions

This study demonstrates a proof-of-concept approach for using ML and NLP to identify PSE reports containing cardiovascular diagnostic error narratives. While the results highlight promising feasibility within a single healthcare system, the generalizability of this approach to other institutions remains unknown. External validation across diverse reporting environments will be essential before considering broader implementation.

## Supporting information

S1 Appendix Appendix A: Hyperparameter optimization. **Appendix B:** List of TF-IDF features. **Appendix C:** Optimized hyperparameters for the XGBoost model. **Appendix D:** List of TF-IDF features selected with Chi-square test. Appendix E: Performance of classifying PSE reports with cardiovascular diagnostic error using patient demographics and TF-IDF features.
(DOCX)
