# Supplementary material for: Applying machine learning and natural language processing to patient safety event reports: Identifying patterns of cardiovascular diagnostic errors
Source: PLoS One. 2026 Apr 10;21(4):e0345693. doi: 10.1371/journal.pone.0345693 (PMC13068243; doi:10.1371/journal.pone.0345693)
Supplement: S1 Appendix — Appendix B: List of TF-IDF features. Appendix C: Optimized hyperparameters for the XGBoost model. Appendix D: List of TF-IDF features selected with Chi-square test. Appendix E: Performance of classifying PSE reports with cardiovascular diagnostic error using patient demographics and TF-IDF features. (DOCX) [file pone.0345693.s001.docx]

**Applying Machine Learning and Natural Language Processing to Patient Safety Event Reports: Identifying Patterns of Cardiovascular Diagnostic Errors**

Azade Tabaie^1,2,*^, Alberta K. Tran^2,3,4^, Codrin Parau^5^, Sonita S. Bennett^1^, Sadaf Kazi^2,5^, Kristen E. Miller^2,5^

^1^Center for Biostatistics, Informatics, and Data Science, MedStar Health Research Institute, Washington, DC

^2^Department of Emergency Medicine, Georgetown University Medical Center, Washington, DC

^3^MedStar Health Research Institute, 3007 Tilden St. NW, Washington, DC

^4^MedStar Health Institute of Quality and Safety, 10980 Grantchester Way, Columbia, MD

^5^National Center for Human Factors in Healthcare, MedStar Health Research Institute, Washington, DC

*Corresponding Author:

Azade Tabaie, PhD

AI Data Scientist, Center for Biostatistics, Informatics, and Data Science, MedStar Health Research Institute

Assistant Professor, Emergency Medicine, Georgetown University Medical Center

3007 Tilden St NW, Washington, DC 20008

Email: Azade.Tabaie@MedStar.net

# **Appendix A**

**Hyperparameter Optimization**

- Elastic net
  - Optimization approach: cross-validation and grid search
  - Range of hyperparameter tested:
    - alpha_values = [1e-3, 1e-2, 1e-1, 0.5, 1, 4, 5, 10, 15, 20]
    - L1_Ratio_values = [0.0001, 0.001, 0.01, 0.1, 0.2, 0.5, 0.7, 0.9, 0.95, 0.99, 1]
- XGBoost
  - Optimization approach: Bayesian optimization
  - Range of hyperparameter tested:
    - learning_rate: (0.01–1)
    - max_depth: (1–5)
    - max_delta_step: (0–5)
    - reg_lambda: (0.1–10)
    - reg_alpha: (0.01–1.0)
  - Stopping criterion: Best AUROC in 50 iterations
- DNN
  - Optimization approach: Bayesian optimization
  - Range of hyperparameter tested:
    - learning_rate: [1e-1, 5e-1, 1e-2, 5e-2, 1e-3, 5e-3, 1e-4, 5e-4, 1e-5, 5e-5]
    - dense_units: [32, 64, 128])
    - dropout layer 1: (0.1, 0.5)
    - dropout layer 2: (0.1, 0.5)
  - Stopping criterion: training stops when the validation loss has not decreased for 3 consecutive epochs. If the validation loss is consistently decreasing, the maximum number of optimization iteration is 50.

# **Appendix B**

**List of TF-IDF Features**

abdomen, abdomin, abnorm, accept, access, acquir, action, activ, actual, acut, addit, address, administ, administr, admiss, admit, advis, agre, air, alert, allow, almost, alter, ama, anesthesia, answer, antibiot, appear, appli, appoint, appropri, approxim, area, arm, arriv, arteri, ask, assess, assign, assist, associ, assum, assur, attach, attempt, attend, avail, await, awar, back, bag, base, bed, bedsid, begin, behavior, believ, bilater, bipap, ble, bleed, blood, blue, board, bolu, bowel, brain, breath, bring, busi, call, came, cancel, cancer, cardiac, cardiolog, cardiologist, care, cathet, caus, ccu, center, central, charg, chart, check, chest, chronic, clarifi, clean, clear, clinic, close, code, cold, collect, come, comfort, commun, complain, complaint, complet, complic, compress, comput, concern, condit, confirm, confus, connect, consent, consid, consist, consult, contact, continu, contraind, contrast, control, convers, coordin, correct, cough, cours, cover, covid, cpr, critic, cta, cultur, current, cva, cxr, daili, decid, decis, declin, decreas, delay, deliv, deni, depart, desk, despit, detail, determin, develop, devic, diabet, diagnos, diagnosi, dialysi, differ, difficult, difficulti, direct, directli, director, discharg, discontinu, discov, discuss, distress, document, dose, draw, drawn, dress, drip, drop, dvt, earli, earlier, eat, echo, educ, effect, ekg, elev, emerg, ensur, enter, episod, error, escal, evalu, even, event, eventu, exam, examin, experienc, explain, express, extravas, extrem, eye, facil, fact, fail, failur, fall, fax, feel, fellow, fever, fill, final, find, fine, finger, flow, fluid, flush, foley, follow, foot, forc, forearm, function, gener, glucomet, glucos, gtt, hand, handoff, hang, happen, hard, harm, head, heart, help, heparin, high, histori, hold, hospit, hospitalist, hypertens, hypoglycemia, hypotens, ice, icu, identifi, imag, imc, immedi, import, improv, inappropri, incid, incorrect, increas, indic, infect, infiltr, inform, infus, initi, inject, injector, injuri, inpati, inquir, insert, insist, instruct, insulin, intern, intervent, intub, investig, involv, issu, lab, lack, lactic, late, later, lead, least, leav, leg, level, like, limit, line, list, locat, long, longer, lovenox, low, lower, machin, manag, mani, manner, mar, mask, measur, medconnect, medic, medicin, member, mental, mention, messag, micu, midnight, miss, monitor, month, morn, move, nausea, necessari, neck, need, neg, neurolog, new, normal, note, notic, notif, notifi, npo, observ, obtain, occur, occurr, offer, offic, old, omni, omnipaqu, open, oper, order, orient, origin, outpati, outsid, overnight, oxygen, pacemak, pacu, paperwork, pass, pelvi, pend, perform, period, person, pharmaci, phone, physic, picc, place, placement, plan, pmh, point, polici, poor, posit, possibl, post, potenti, power, prbc, precaut, prepar, present, pressur, prevent, prior, problem, procedur, proceed, process, progress, proper, properli, protocol, provid, pse, pui, pull, puls, pump, push, put, question, quickli, radiat, radiolog, radiologist, rapid, rate, reach, reaction, read, readi, realiz, reason, receiv, recent, recheck, recommend, record, red, refer, refus, relat, relay, remain, remov, repeat, replac, repli, report, request, requir, resid, resolv, resourc, respiratori, respond, respons, rest, restart, return, reveal, review, risk, room, round, routin, rrt, rule, run, safe, safeti, salin, sat, satur, sbp, scan, scd, schedul, screen, secretari, sedat, see, seem, seizur, send, sepsi, set, sever, shift, short, shortli, show, sicu, side, sign, signific, sit, site, situat, skin, small, sob, soon, speak, specif, spine, spo, stabl, staff, stand, standard, start, stat, state, statu, stay, step, stick, still, stop, stress, stretcher, stroke, studi, subsequ, success, sugar, suggest, supervisor, suppos, sure, surgeon, surgeri, surgic, suspect, swallow, swell, switch, symptom, system, tabl, task, team, technician, technologist, tele, telemetri, tell, temperatur, test, thank, therapi, therapist, think, though, time, today, total, tower, tpa, transfer, transfus, transit, transplant, transport, trauma, treat, treatment, tri, triag, troponin, tube, turn, twice, type, ultim, ultrasound, unabl, unawar, understand, underw, unit, unknown, unrespons, unsaf, updat, upset, urgent, urin, valu, vascular, ventil, verbal, verifi, view, visit, vital, vomit, vte, wait, walk, want, warm, way, weak, wheelchair, worsen, wound, wrong, xray

# **Appendix C**

**Optimized Hyperparameters for the XGBoost Model**

- Learning_rate = 0.13316950024728014
- Max_delta_step = 0
- Max_depth = 4
- Reg_alpha = 0.8417310404442823
- Reg_lambda = 2.9810824157188547
- Objective = 'binary:logistic'
- N_boosting_rounds = 200

# **Appendix D**

**List of TF-IDF Features Selected with Chi-square Test**

abdomen, abdomin, access, acquir, acut, admiss, admit, advis, agre, antibiot, appli, appropri, area, arm, arriv, arteri, ask, assur, attempt, awar, bag, bed, behavior, bipap, bleed, blue, brain, cancel, cancer, cardiac, cardiolog, cardiologist, central, charg, chart, chest, clean, clinic, close, code, collect, complain, concern, connect, consent, consist, contraind, contrast, coordin, correct, cough, cours, cover, cpr, critic, cta, cultur, cxr, desk, despit, determin, dialysi, dose, dress, echo, educ, ekg, elev, enter, episod, error, event, examin, explain, fail, feel, final, fine, finger, flow, fluid, flush, foley, forc, forearm, glucomet, glucos, gtt, hand, heart, hold, hospit, hypertens, hypoglycemia, hypotens, ice, icu, imag, import, incid, indic, infiltr, initi, inject, injector, inquir, insert, insulin, involv, lab, lactic, lead, leav, level, longer, lovenox, low, machin, mask, monitor, nausea, necessari, neg, notic, obtain, occurr, old, omni, omnipaqu, order, overnight, oxygen, pacemak, pacu, pelvi, perform, pharmaci, picc, plan, pmh, point, power, prbc, precaut, pressur, protocol, provid, puls, question, radiologist, rapid, reaction, readi, recheck, red, refer, relay, remain, remov, repeat, respiratori, respons, return, room, satur, sbp, scan, scd, schedul, see, seizur, sever, shift, situat, skin, small, stand, start, stat, state, step, stick, stop, stress, sugar, swallow, swell, task, technician, tele, telemetri, temperatur, test, thank, therapi, therapist, think, today, tower, transfus, treat, tri, triag, troponin, tube, twice, underw, unrespons, unsaf, urin, vascular, verifi, visit, vomit, vte, wait, walk, want, warm, wound

**Table SC-1. Performance of Classifying PSE Reports with Cardiovascular Diagnostic Error Using Chi-square Selected TF-IDF Features.**

| **Metrics** | **Simple Logistic Regression** | | **Elastic Net** | | **SVM** | | **XGBoost** | | **DNN** | |
| --- | --- | --- | --- | --- | --- | --- | --- | --- | --- | --- |
|  | **Train** | **Test** | **Train** | **Test** | **Train** | **Test** | **Train** | **Test** | **Train** | **Test** |
| **AUROC** | 0.778 | 0.75 | 0.91 | 0.892 | 0.909 | 0.88 | 0.956 | 0.895 | 0.941 | 0.899 |
| **Sensitivity** | 1 | 1 | 0.8 | 0.733 | 0.8 | 0.746 | 0.801 | 0.642 | 0.8 | 0.724 |
| **Specificity** | 0 | 0 | 0.863 | 0.861 | 0.882 | 0.865 | 0.953 | 0.945 | 0.918 | 0.911 |
| **PPV** | 0.155 | 0.155 | 0.517 | 0.491 | 0.555 | 0.503 | 0.758 | 0.683 | 0.642 | 0.6 |
| **NPV** | 0 | 0 | 0.959 | 0.946 | 0.96 | 0.949 | 0.963 | 0.935 | 0.962 | 0.947 |
| **Accuracy** | 0.155 | 0.155 | 0.853 | 0.841 | 0.869 | 0.846 | 0.93 | 0.898 | 0.9 | 0.882 |
| **F-1 Score** | 0.268 | 0.269 | 0.628 | 0.588 | 0.655 | 0.601 | 0.779 | 0.662 | 0.712 | 0.656 |
| **AUPRC** | 0.729 | 0.693 | 0.719 | 0.698 | 0.727 | 0.701 | 0.857 | 0.731 | 0.806 | 0.722 |

# **Appendix E**

The demographic features were age, sex, race, and primary language.

**Table SD-1. Performance of Classifying PSE Reports with Cardiovascular Diagnostic Error Using Patient Demographics and TF-IDF Features.**

| **Metrics** | **XGBoost** | |
| --- | --- | --- |
|  | **Train** | **Test** |
| **AUROC** | 0.988 | 0.915 |
| **Sensitivity** | 0.8 | 0.608 |
| **Specificity** | 0.992 | 0.983 |
| **PPV** | 0.948 | 0.87 |
| **NPV** | 0.964 | 0.932 |
| **Accuracy** | 0.962 | 0.925 |
| **F-1 Score** | 0.868 | 0.716 |
| **AUPRC** | 0.948 | 0.784 |
